# Translating, Contexting, and Institutionalising Knowledge Translation Practices in Northern Australia: Some Reflections: Comment on "Sustaining Knowledge Translation Practices: A Critical Interpretive Synthesis"

**DOI:** 10.34172/ijhpm.2023.7587

**Published:** 2023-04-26

**Authors:** Alexandra Edelman, Stephanie M. Topp

**Affiliations:** College of Public Health, Medical and Veterinary Sciences, James Cook University, Townsville, QLD, Australia

**Keywords:** Knowledge Translation, Health System, Northern Australia, Rural, Remote

## Abstract

In this commentary, we reflect on how the three processes of *translating*, *contexting*, and *institutionalising* knowledge translation (KT) practices, as introduced in a critical interpretive synthesis on sustaining KT, might be drawn on to improve KT sustainability in the northern Australian health system, and some likely challenges. The synthesis provides a useful reminder that health systems are *social* systems and offers an analytical framework against which to map approaches that aim to align knowledge production and utilisation. By positioning "places" of knowledge utilisation and actor roles and networks as key to KT sustainability, the framework also offers the potential to draw attention to non-clinical settings, actors, and relationships that are central to improving health, but that may be historically neglected in KT research and scholarship.

## Introduction

 In their critical interpretive synthesis on sustaining knowledge translation (KT) practices, Borst and colleagues^1^ critique approaches to KT practice and scholarship that approach the concept of “sustainability” as an end-state; ie, the terminus of KT work. Instead, the authors define sustainability of KT practices as ongoing work to improve the use of health research in policy and practice, with an emphasis on *how* states of sustainability are achieved and maintained.^1^ The work therefore provides a useful reminder that health systems are *social* systems; that is, they are comprised of values, norms, ideas, and relationships that shape behaviour and ultimately health system performance, and are continually evolving and morphing as a result.^2^

 The authors propose that researchers use three concepts to frame their conceptual and empirical work on KT with sustainability in mind: *translating*, *contexting*, and *institutionalising*. These concepts are introduced and explained as essential processes that underpin the work needed for sustainability of KT practices within health systems. In this Commentary, we reflect on how the three processes of *translating*, *contexting*, and *institutionalising* KT practices might be drawn on to improve KT sustainability in the northern Australian health system, and some likely challenges.

## Translating, Contexting, and Institutionalising Knowledge Translation Practices in Northern Australia

 Northern Australia’s vast and rural and remote geography — 1.3 million people living over 3 million km^2^ — and proximity to the Asia Pacific region shape a distinctive set of health service characteristics. Around 30% of Aboriginal, and Torres Strait Islander, Australians live in northern Australia and there are over 200 distinct Aboriginal, and Torres Strait Islander, communities across the north.^3^ Key health service strengths include distributed models of care and workforce training, Aboriginal and Torres Strait Islander community-led primary care services and tertiary healthcare facilities in larger regional centres. Persistent challenges, however, include health workforce shortages, long distances between services, service fragmentation and under-resourcing of public health and preventive services.^4^ Alongside adverse social determinants of health, health service and workforce gaps contribute to higher rates of both chronic and infectious diseases with disparities increasing with remoteness.^4^ In addition, connection to culture and country is a key factor shaping Aboriginal, and Torres Strait Islander, health and wellbeing, yet many services and service models fail to deliver culturally safe care close to home.^3,5^ Against this backdrop, there is a growing interest, and organisational commitment towards, KT-focussed initiatives concerned with health equity — encouraged by new government investments in KT and innovation.^6^

 Borst et al^1^ define *translating* processes as work to create “networks between knowledge producing communities and actors that may be seen as intended users of such knowledge, and a mutually adaptive process where both the knowledge and its supposed utilisation environment are aligned with each other.” This definition sees translation as both the transformation of knowledge to make it utilisable (as commonly seen in definitions of KT^7^), *and* as creating connections between KT actors and “places” of knowledge utilisation. In other words, not only does KT require production and dissemination of new knowledge in ways that enhance its utility to end-users (eg, clinicians, health service administrators, policy-makers), it requires ongoing work to foster connections between KT actors to assign roles and mobilise action. The concept of *translating* thus highlights that knowledge production activities should be framed by an understanding of what actions, led by whom, will drive practice/policy change in response to evidence, and who will benefit from these actions. Numerous northern-led projects demonstrate the benefits to patients and communities of creating strategic partnerships between local actors to align knowledge production and utilisation^8^; and it is helpful that the synthesis provides an analytical framework against which to map this approach and explore the challenges to KT in specific settings. The framing of research knowledge use in policy and practice as necessarily iterative and dynamic underscores the need to actively foster relationships between researchers and end users throughout data-to-knowledge-to-practice-to-data cycles. This approach challenges linear models of research production and use that tend to overlook *how* and *by whom *new knowledge will be implemented in real world contexts. A growing body of literature advocates the use of complexity and network principles to support such efforts.^9^

 Practically, the capacity to create and sustain productive KT networks in northern Australia is challenged by health workforce issues — including high workforce turnover, shortages, and reliance in many remote areas on fly-in-fly-out service models to fill workforce gaps. The emphasis in the synthesis on relationships as pivotal to sustaining KT practices draws attention to the urgent imperative to strengthen the local health workforce to create a foundation for sharing new ideas and co-producing knowledge. The *translating* concept also underscores that investments in research-related skills, and in time for research, are needed to provide opportunities for clinicians and health service administrators to lead and participate in research close to practice and planning. Key “places” of knowledge utilisation in northern Australia include primary care and community settings in rural and remote areas; it is therefore essential that research agendas are driven by locally based community members, clinicians, planners, and researchers who can actively create local service improvement and health benefits through research.^10^ Such locally-driven and problem-led approaches require deliberate investments in strengthening the health workforce, building research skills, and resourcing collaborative research efforts focussed on health equity — yet the north has suffered from longstanding underinvestment in these areas.^3^

*Contexting* processes, as defined in the synthesis, involve constantly constructing contexts that “knit” actors together in networks that support and sustain KT practices.^1^ This concept challenges researchers to proactively identify what is needed to create networks to support KT practices, which contrasts with research approaches that consider content (eg, a clinical or health service intervention) and context separately. Understanding and incorporating context in research is necessary in efforts to drive policy and practice change, such as evidence-informed health service reform. For example, Aboriginal cultural ways of knowing and doing, and Aboriginal leadership and community ownership across the research process, is fundamental to research that aims to improve Aboriginal health and wellbeing.^11^ Indigenist and decolonising research approaches that foreground Indigenous ways of knowing, being, and doing^12^ recognise that research practice is inseparable from social, economic and cultural impacts in a particular setting and therefore necessitates a much more collaborative approach.

 The concept of *contexting* may empower “KT actors” (whether researchers, clinicians, or administrators) to proactively map and consider how societal impacts from their work might be enabled by change and reform in their organisational setting and broader health system; but for many individuals this is likely to be an unfamiliar approach because of positivist research traditions and siloed thinking. For example, in northern Australia, the co-existence of multiple funding buckets with separate performance indicators and reporting lines between, and even within, health service organisations incentivises narrow metrics-driven approaches to planning.A key challenge, then, is that KT actors in this environment may not even recognise themselves as being so influenced, let alone actively construct contexts that bring people together to drive change in response to evidence.

*Contexting* is also likely to be particularly challenging in settings, such as in northern Australia, where organisational and regulatory health system features that mediate against KT practices are difficult to address locally. For example, a lack of incentives for research in health service funding agreements in northern Queensland detracts from efforts to embed KT practices in health services.^13^ If structural health system elements such as funding and performance indicators are poorly aligned with KT practices, whose role is it to identify and address these impediments to construct more conducive arrangements for KT? And how can rurally-based researchers, clinicians, academics or health service administrators routinely include urban-based policy-makers and funders in local level *contexting* processes to explore and enact requisite policy changes? While *contexting*is essential to coordinate KT practices across organisations to ultimately improve healthcare and outcomes in northern Australia, addressing underpinning structural flaws in healthcare financing and governance is notoriously challenging and political.^14^ Successful *contexting*, therefore, requires bodies of knowledge that explain how health systems operate and change — including how constituent “hardware” (ie, finance, medical products, information systems, services, and workforce) and “software” (ie, ideas, interests, values, norms, and power dynamics) components underpin health system performance.^15^

 The third essential process needed for sustainability of KT practices is *institutionalising*. As defined in the synthesis, *institutionalising* processes refer to “the strategic use of institutions as to create a (temporary) fundament on which KT practices can be organised.”^1^ This concept highlights how institutions (such as academia, medicine, or advocacy groups) and infrastructures (such as physical spaces, organisations or agreements) can be used by KT actors to create and sustain KT practices. The logic of institutionalising is therefore that individuals can draw on institutions and infrastructures to support and convey legitimacy on their work. *Institutionalising* processes may be particularly useful for researchers, clinicians, community members, and policy-makers in geographically distributed settings, such as northern Australia, to pursue objectives where there are fewer opportunities for in-person interactions between KT actors, smaller communities of practice, and greater distances from centres of decision-making power and influence. Rural generalism, for example, has developed as a powerful mobilising force against medical specialisation. A workforce strategy developed in Queensland to support junior doctors train and develop careers in rural and remote medicine, rural generalism has contributed to addressing workforce shortages outside of metropolitan and regional practice settings and presents a fundament to champion equity in healthcare.^16^

 Government-incentivised efforts to support *institutionalising* in northern Australia include the establishment of academic health centres Australia-wide as new organisations that aim to create cross-organisational partnerships, supported by a dedicated governance structure and funding, to improve collaboration for KT in healthcare.^17^ However, it remains to be seen how KT actors are strategically using these entities to create and sustain KT practices.^18^ Creating a new structure to support collaboration around shared high-level goals is unlikely by itself to change how people work together, and regulative, normative and cultural-cognitive forces contributing to goal realisation need to be considered.^19^ As noted earlier, any health system intervention and reform must take account of both “hardware” and “software” elements to be successful and sustained.

## The Importance of Conceptual Clarity and Cross-pollination of Ideas

 Throughout the synthesis, Borst et al^1^ demonstrate the “conceptual unclarity” and contested nature of several KT-related terms that are used by researchers, practitioners, and policy-makers (including “translation,” “sustainability,” “context,” and “institution”). Against an often murky and contested conceptual landscape, the authors offer much-needed clarity by highlighting, within the definitions of the three key concepts, the critical role of actors, networks, and relationships in shaping health system performance. A key contribution of the synthesis, therefore, is its deliberate effort to conceptually combine research fields, which involved imbuing technocratic, linear definitions of key terms with ideas from actor-network theory. The *institutionalising* concept, too, offers an important contribution to the institutional theory field which has tended to neglect analysis and critique of power structures and relations.^20^

 As the synthesis authors argue, a cross-pollination of ideas across research fields is critical to achieve KT goals.^1^ To that end, and recognising the continual challenge of elevating public and population health concerns in research contexts intent on clinical medicine, we suggest that an integrated, whole-of-health system perspective (as supported by systems thinking approaches^21^ or adoption of complex adaptive systems principles^22^) will be important to investigate how processes of *translating, contexting, and institutionalising* can be directed towards improving population health, including to improve pandemic preparedness and response. A population health lens is critical because the primary purpose of health systems is to improve health, which includes health improvement activities (eg, healthcare) as well as prevention of poor health and disease, including efforts to influence the social and environmental determinants of health.^23^ By positioning “places” of knowledge utilisation and actor roles and networks as key to KT sustainability, the framework offers the potential to draw attention to non-clinical settings, actors, and relationships that may be historically neglected in KT research and scholarship.

## Ethical issues

 Not applicable.

## Competing interests

 Authors declare that they have no competing interests.

## Authors’ contributions

**Conceptualization: **Alexandra Edelman.

**Writing–original draft:** Alexandra Edelman.

**Writing–review & editing:** Alexandra Edelman and Stephanie M. Topp.

## Funding

 SMT holds a NHMRC Investigator Award (GNT1173004).
